# Enhanced protection to *Mycobacterium tuberculosis* infection in IL-10-deficient mice is accompanied by early and enhanced Th1 responses in the lung

**DOI:** 10.1002/eji.201040433

**Published:** 2010-06-01

**Authors:** Paul S Redford, Andre Boonstra, Simon Read, Jonathan Pitt, Christine Graham, Evangelos Stavropoulos, Gregory J Bancroft, Anne O'Garra

**Affiliations:** 1 Division of Immunoregulation, MRC National Institute for Medical ResearchThe Ridgeway, Mill Hill, London, UK; 2 Department of Gastroenterology and Hepatology, Erasmus Medical CenterRotterdam, The Netherlands; 3 Biopharmaceuticals Research UnitMaløv, Denmark; 4 Immunology Unit, Department of Infectious & Tropical Diseases, London School of Hygiene and Tropical MedicineKeppel Street, London, UK

**Keywords:** Granulocytes, IL-10, IL-17, *Mycobacterium tuberculosis*, Th1

## Abstract

IL-10 regulates the balance of an immune response between pathogen clearance and immunopathology. We show here that *Mycobacterium tuberculosis* (*Mtb*) infection in the absence of IL-10 (IL-10^−/−^ mice) results in reduced bacterial loads in the lung. This reduction was preceded by an accelerated and enhanced IFN-γ response in the lung, an increased influx of CD4^+^ T cells into the lung, and enhanced production of chemokines and cytokines, including CXCL10 and IL-17, in both the lung and the serum. Neutralization of IL-17 affected neither the enhanced production of CXCL10 nor the accumulation of IFN-γ-producing T cells in the lungs, but led to reduced numbers of granulocytes in the lung and reduced bacterial loads in the spleens of *Mtb*-infected mice. This suggests that IL-17 may contribute to dissemination of *Mtb*.

## Introduction

Tuberculosis (TB) is primarily a lung disease, and dissemination of the pathogen depends on productive infection of this critical organ 1–3. A major role for CD4^+^ T cells in protection against *Mycobacterium tuberculosis* (*Mtb*) infection is well documented 1–3. To eradicate a pathogen such as *Mtb*, the host must mount a protective response, which must be strictly regulated to limit pathology while avoiding chronic infection.

A role for IL-12 and the Th1 cytokine IFN-γ is established in protection against mycobacterial infections in both mouse models 4, 5 and human disease 6. Early influx of T cells producing IFN-γ has been demonstrated in *Mtb* resistant as compared with more susceptible mouse strains 7. Vaccination triggers an IL-17-dependent accelerated IFN-γ response by CD4^+^ T cells in the lung seen upon *Mtb* infection 8. Although produced by CD4^+^ 8 and γδ T cells 9 during *Mtb* infection, IL-17 has been shown to have a limited role in host defense 10, 11. TNF is critical for control of *Mtb* infection in both mouse and man 2, 12, 13. IFN-γ-mediated macrophage activation together with TNF is required in the control of mycobacterial growth 4, 5, 12, at least in part by the induction of nitric oxide 14, both cytokines being involved in innate and adaptive immune protective responses to *Mtb* infection.

Since IL-10 suppresses macrophage and DC functions, including killing of intracellular pathogens and TNF and IL-12 production required for Th1 responses 15, 16, it is likely that IL-10 induction during *Mtb* infection may affect the course of disease. IL-10 may inhibit immunopathology as seen during infections with intracellular pathogens 17, 18, or curb pathogen clearance contributing to chronic infection 19–22. In support of a role for IL-10 in control of immune responses to mycobacterial infection, IL-10 mRNA is induced during experimental infection with a number of mycobacterial species, including *Mtb* 23*,* and has been correlated with enhanced disease in TB patients 24–27. Although IL-10-deficient (IL-10^−/−^) mice infected with *M. avium* and *M. bovis* bacillus Calmette–Guerin 28–31 show enhanced mycobacterial clearance, reports regarding a role for IL-10 in limiting protection to *Mtb* infection have been conflicting and inconclusive 23, 28, 32–35.

Upon aerosol infection with *Mtb*, the acquired cellular response is slow to be induced in the lung. *Mtb* may infect diverse types of phagocytic cells 36, 37. The dissemination of mycobacteria from the lung to the draining LN (dLN) has been suggested to involve lung DC 36–41, leading to the activation of antigen-specific T cells and the induction of effector function 42–44. Although IL-12p40 promotes DC migration during mycobacterial infection 41, IL-10 may limit it 39.

We show here that IL-10 does indeed control the immune response to *Mtb* infection. Our findings show that infected IL-10^−/−^ mice maintain a reduced bacterial load in lungs with decreased dissemination to the spleen, which is preceded by an earlier and enhanced Th1-type response in the lung. In contrast, IL-17 appeared to enhance dissemination to the spleen during primary pulmonary *Mtb* infection.

## Results

### Enhanced control of *Mtb* infection in IL-10^−/−^ mice

**Figure 1:**
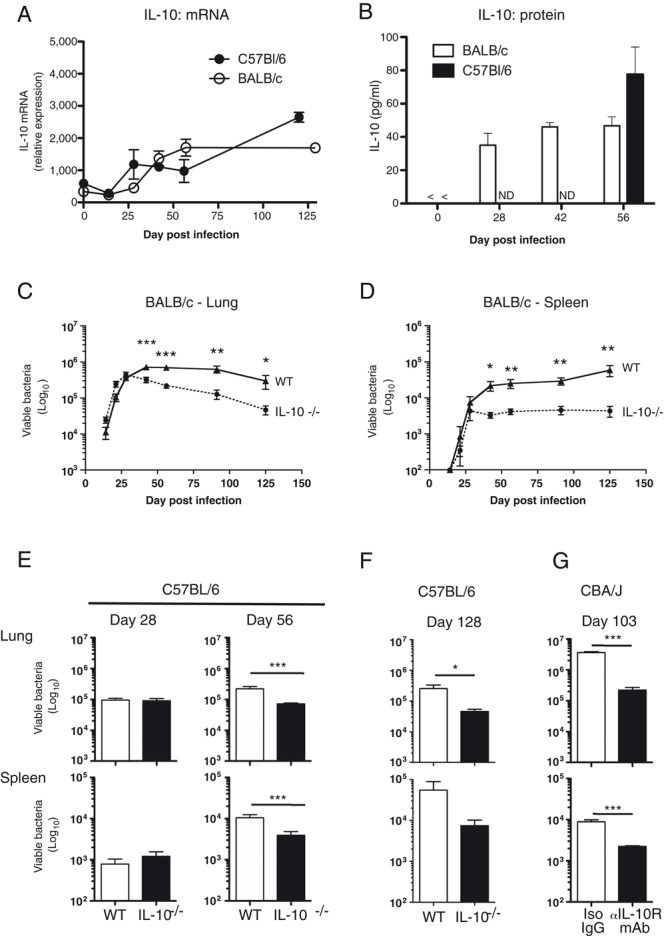
IL-10 limits bacterial clearance following aerosol infection with Mtb. (A) *Il10* mRNA expression (RT-PCR) and (B) IL-10 protein (ELISA) following *ex vivo* restimulation with PPD of lung cell homogenates prepared from aerogenically *Mtb*-infected BALB/c and C57BL/6 mice. (C, D) WT BALB/c and BALB/c IL-10^−/−^ mice were killed at the time points indicated postinfection and bacterial burdens were determined in the (C) lungs and (D) spleen. The BALB/c data shown here are combined from six independent experiments. (E, F) Bacterial burdens in the lungs and spleens of WT (open bars) and IL-10^−/−^ (closed bars) C57BL/6 mice were determined at the time points indicated. Data from C57BL/6 mice are (E) combined from two independent experiments (*n*=11–15 mice *per* group) and (F) from one independent experiment (*n*=7 mice *per* group). (G) CBA/J mice were treated before and during *Mtb* infection with anti-IL-10R mAb (or control IgG) and bacterial burdens determined at day 103 postinfection (data are from one independent experiment, *n*=5–6 mice *per* group). (Data are mean±SEM). ^*^p<0.05; ^**^p<0.01; ^***^p<0.001 (unpaired Student's *t*-test); ND, not done; <, below detection limit of the assay.

To explore a potential role of IL-10 in the control of the immune response to *Mtb H37Rv*, we first investigated the expression of this cytokine in the lungs of mice upon aerogenic infection. The expression of IL-10 mRNA (Fig. 1A) and IL-10 protein (Fig. 1B) was induced following *Mtb* infection in the lungs of BALB/c and C57BL/6 in accordance with the previous reports 23. IL-10^−/−^ mice showed a significant and prolonged tenfold reduction in bacterial load in lungs and spleen from day 28 postinfection until the end of the study, as compared with WT control mice (Fig. 1C and D). These effects were not restricted to mice on the BALB/c background, as the absence of IL-10 in mice on C57BL/6 background (Fig. 1E and F), or neutralization of its signalling capacity by mAb in CBA/J mice (Fig. 1G), significantly reduced bacterial burdens in the lungs and spleen at the time points investigated, as reported previously 34.

### Enhanced and accelerated Th1 responses in lungs of infected IL-10^−/−^ mice

**Figure 2:**
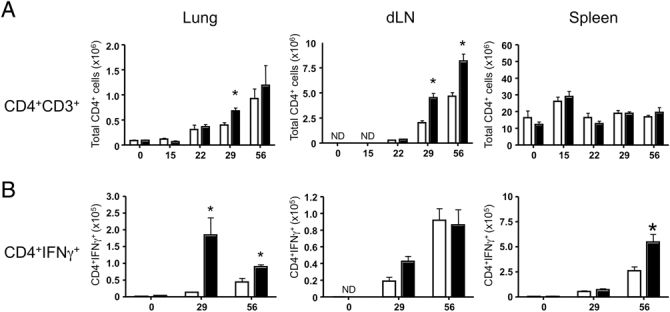
Earlier and enhanced accumulation of CD4^+^ T cell and cytokine-producing CD4^+^IFNγ^+^ cells in the lungs of infected IL-10^−/−^ as compared with WT mice. (A) The total number of CD4^+^CD3^+^ T cells present in the indicated organs of WT (open bars) and IL-10^−/−^ (closed bars) BALB/c mice infected with *Mtb* was determined by flow cytometry and calculated based on the total cell counts acquired at each time point for each individual mouse. The percentage of CD4^+^IFNγ^+^ T cells (Supporting Information Fig. 1B) and total numbers of CD4^+^IFNγ^+^ cells (B) were determined following restimulation of whole organ homogenates *ex vivo*. Cells were stained with the appropriate Ab and CD4^+^IFNγ^+^ levels determined by flow cytometry according to the gating strategy shown in Supporting Information Fig. 1A. Data are mean±SEM and are representative of at least two independent experiments (*n*=3–4 mice *per* group). ^*^p<0.05 (unpaired Student's *t*-test); ND, not done.

To determine whether enhanced protection against *Mtb* infection reflected an accelerated and/or enhanced immune response to the pathogen, cell suspensions from lungs, dLN and spleens were analysed. An increase in the numbers of CD4^+^ T cells (Fig. 2A) was observed in the lungs and dLN of IL-10^−/−^ mice at day 29 after infection with *Mtb* as compared with controls. This increase in CD4^+^ T-cell numbers was not observed in spleens from infected IL-10^−/−^ mice (Fig. 2B). Furthermore, increased percentages of IFN-γ^+^ CD4^+^ T cells were observed in lungs of IL-10^−/−^ mice infected with *Mtb* at all time points after infection, with the most striking difference apparent at day 29 (0.91%, WT to 4.83%, IL-10^−/−^ mice: Supporting Information Fig. 1B). In contrast, increased percentages of IFN-γ-producing CD4^+^ T cells in IL-10^−/−^ mice were not observed in the dLN or minimally enhanced in the spleen but only much later after infection as compared with WT mice. The increased numbers of CD4^+^ T cells (Fig. 2A) and percentages of CD4^+^ IFN-γ-producing T cells were observed early in the lung after *Mtb* infection of IL-10^−/−^ mice, translated to highly significant increases in the numbers of Th1 cells (Fig. 2B). Collectively, these data suggest that IL-10^−/−^ mice show significant and sustained *Mtb* control, which correlated with an accelerated and enhanced expansion of CD4^+^ T cells in the dLN, together with increased differentiation, expansion and migration of Th1 cells to infected lungs (Fig. 2B).

**Figure 3:**
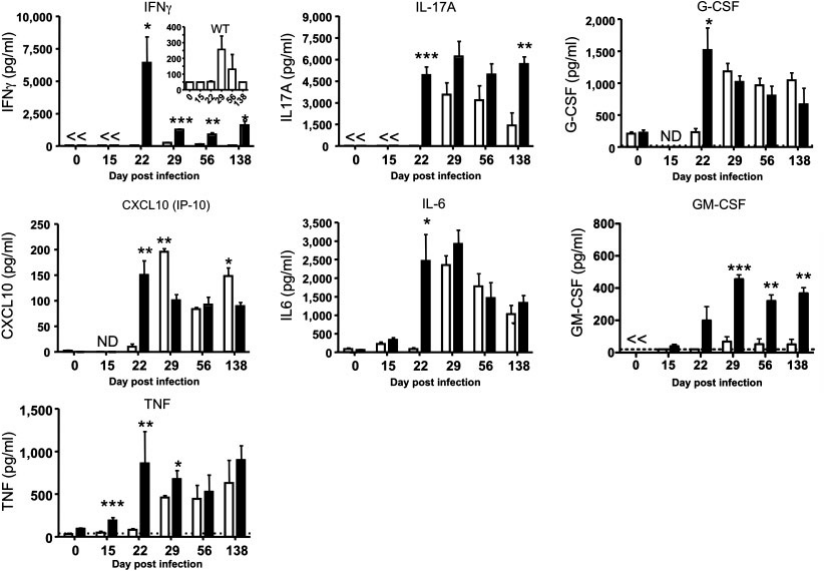
Following Mtb infection, IL-10^−/−^ mice have an earlier and enhanced production of proinflammatory cytokines in the lungs as compared with WT mice. Lung tissue from *Mtb-*infected WT (open bars) and IL-10^−/−^ BALB/c (closed bars) mice was isolated, homogenized and restimulated with PPD after which supernatants were removed and screened by Multiplex assay for the indicated cytokines (see also Supporting Information Table 1). Data are mean±SEM and are representative of at least two independent experiments (*n*=3–4 mice *per* group). ^*^p<0.05; ^**^p<0.01; ^***^p<0.001 (unpaired Student's *t*-test); ND, not done; <, below detection limit of the assay.

**Figure 4:**
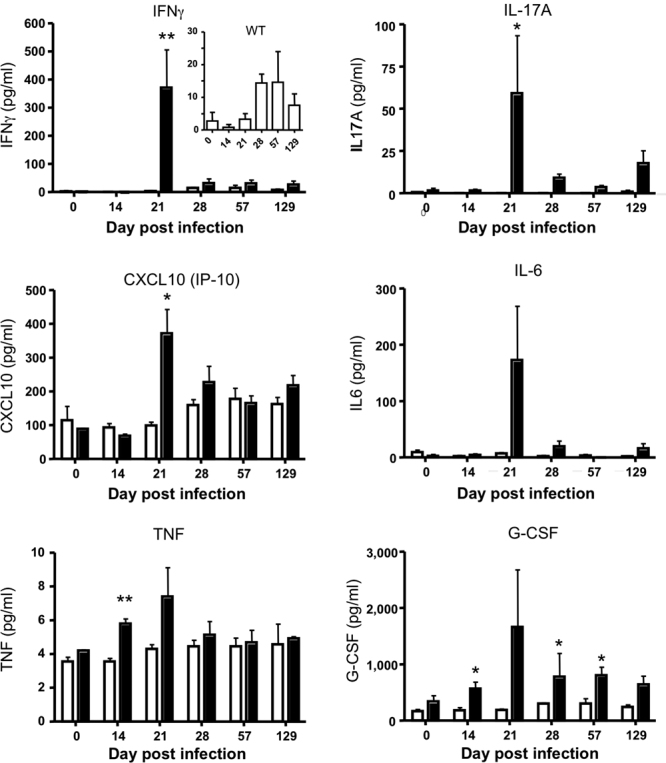
IL-10^−/−^ mice have enhanced levels of proinflammatory mediators in the serum, following Mtb infection as compared with WT mice. WT BALB/c (open bars) and IL-10^−/−^ (closed bars) BALB/c mice were infected with *Mtb*. At specific time points, postexposure groups of mice were killed, serum was obtained from the coagulated blood samples and screened by a multiplex cytokine profiler assay (Supporting Information Table 2). The data are mean±SEM and are representative of two independent experiments (*n*=3–5 mice *per* group). ^*^p<0.05; ^**^p<0.01 (unpaired Student's *t*-test).

To further investigate the local immune response to *Mtb* infection, lung cell suspensions were restimulated with purified protein derivative (PPD) and cytokine/chemokine production was evaluated in supernatants, initially performing a primary screen on 22 analytes, using a multiplex cytokine assay (Fig. 3 and Supporting Information Table 1) at a chosen time point (day 28) based on the preliminary data. In keeping with the enhanced numbers of Th1 cells in the lungs of infected IL-10^−/−^ mice (Fig. 2B), the levels of IFN-γ were significantly elevated early after *Mtb* infection in supernatants from IL-10^−/−^ restimulated lung suspensions (Fig. 3). This was accompanied by increased levels of CXCL10 (IP-10), TNF, IL-17A, IL-6, G-CSF, and to a lesser extent GM-CSF, which all peaked at day 22 postinfection (Fig. 3). Increases in IL-1α, IL-1β, IL-2 and CCL3 were seen upon infection with *Mtb* and elevated to a small extent in supernatants from infected IL-10^−/−^ mice *versus* controls; little to no change was seen in supernatants of IL-12p70, IL-4, IL-5, IL-7, IL-9 and IL-13 upon *Mtb* infection and no difference between WT and IL-10^−/−^ mice – any small differences were variable (Supporting Information Table 1). Differences between WT and IL-10^−/−^ mice were not observed for CXCL10, TNF, IL-6 and G-CSF by day 56 postinfection. In contrast, elevated levels of IFN-γ, IL-17A and GM-CSF were still apparent at day 56 postinfection in supernatants (Fig. 3). To further support this hypothesis, using the multiplex assay, similar trends in IFN-γ, CXCL10, TNF, IL-17A, IL-6 and G-CSF only were also observed in sera from IL-10^−/−^ mice albeit at much lower levels (Fig. 4; Supporting Information Table 2). In accordance with our findings in the lung (Fig. 3), each cytokine/chemokine in the serum of IL-10^−/−^ mice peaked at around 21 days after infection, suggesting that elevated levels in the sera resulted from spill-over of the immune response to the pathogen in the infected lung. Thus the capacity of IL-10^−/−^ mice to respond to *Mtb* infection with increased and earlier production of IFN-γ, CXCL10, IL-17A, IL-6, G-CSF and GM-CSF in the lung (Fig. 3) and serum (Fig. 4) correlates with a subsequent, pronounced and long-lasting significant decrease in bacterial load (Fig. 1C and D).

### Th1 responses in lungs of *Mtb* infected IL-10^−/−^ mice are IL-17 independent

**Figure 5:**
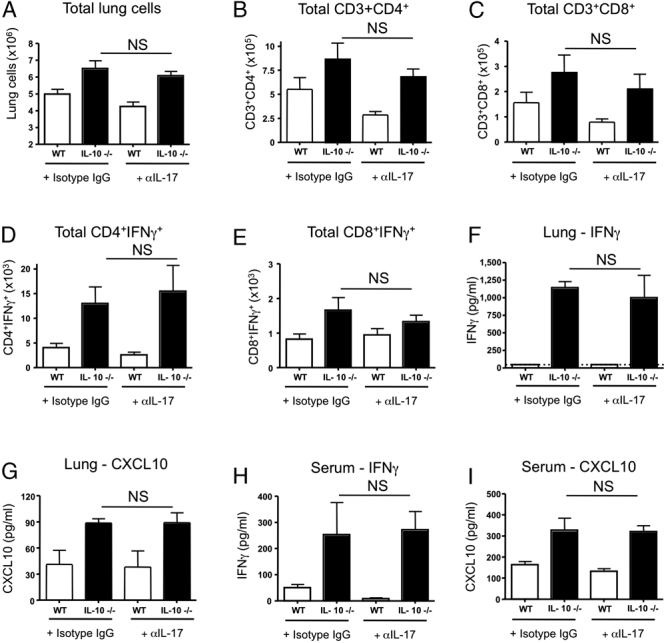
The enhanced cellular influx and IFN-γ production seen in IL-10^−/−^ mice during *Mtb* infection are not IL-17 dependent. WT (open bars) and IL-10^−/−^ (closed bars) BALB/c mice were infected with *Mtb* and treated twice weekly with either anti-IL-17 mAb or isotype IgG. Mice were killed on day 32 postinfection and the lungs isolated. (A) Total lung cell counts, (B) total CD4^+^ T cells and (C) total CD8^+^ T cells following IL-17 neutralization were determined by flow cytometry staining for CD3, CD4 and CD8. Whole lung homogenates from anti-IL-17-treated and isotype control-treated mice were restimulated with PPD and the total number of (D) CD4^+^IFN-γ^+^ and (E) CD8^+^IFN-γ^+^ cells in the lungs of infected WT and IL-10^−/−^ BALBc mice was determined by flow cytometric analysis. Cell culture supernatants of the restimulated lung homogenates were analysed for (F) IFN-γ and (G) CXCL10. Serum levels of (H) IFN-γ and (I) CXCL10 from *Mtb*-infected Ab-treated mice were assayed as described in Fig. 4. Results (mean±SEM) shown are combined data from two independent experiments, totalling ten mice *per* group (A–E) or three to six mice *per* group (F–I). NS, nonsignificant (unpaired Student's *t*-test).

Our data suggest that the enhanced differentiation and early influx of Th1 cells producing IFN-γ into the lungs of IL-10^−/−^ mice infected with *Mtb* results in the subsequently enhanced bacterial clearance. The elevated levels of IL-17A (Fig. 3) and IL-17A-producing cells (Supporting Information Fig. 2A–D) that we also observe in the lungs of infected IL-10^−/−^ mice could account for the enhanced production of the chemokine CXCL10 (Fig. 3) and the early influx of Th1 effector cells into the lung (Fig. 2B), as previously reported following vaccination 8. Alternatively, deficiency of IL-10 in our system could bypass the requirement for IL-17A and CXCL10 production and enhanced Th1 influx into the lung, resulting in subsequent protection against *Mtb*. To test this, IL-17 was neutralized in mice *via* use of a mAb before and during infection with *Mtb*, and effects on IFN-γ and CXCL10 production and cellular influx into the lung were examined. The enhanced cellular influx, including that of CD4^+^ and CD8^+^ T cells, observed in *Mtb* infected IL-10^−/−^ mice as compared with WT control mice was unaffected by neutralization of IL-17 (Fig. 5A–C). Furthermore, the increased numbers of IFN-γ-producing CD4^+^ and CD8^+^ T cells (Fig. 5D and E), and the increased amounts of IFN-γ and CXCL10 seen in supernatants of stimulated lung cells (Fig. 5F and G), or in serum (Fig. 5H and I) or in infected IL-10^−/−^ mice were not significantly affected following IL-17 neutralization. Hence, our findings suggest that, in contrast to vaccination 8, an absence of IL-10 can enhance and accelerate Th1 responses in the lung during primary *Mtb* infection independently of IL-17.

### Effects of neutralizing IL-17 during primary *Mtb* infection

**Figure 6:**
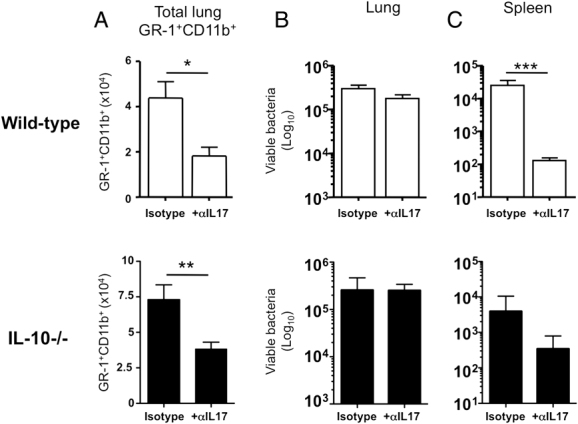
The presence of IL-17 during *Mtb* infection in mice has adverse effects on bacterial dissemination to secondary disease sites. WT (open bars) and IL-10^−/−^ BALB/c (closed bars) mice were treated with anti-IL-17 Ab (or isotype control IgG) before and during infection with *Mtb*. Mice were killed on day 32 postinfection and the lungs were isolated, homogenized and analysed by flow cytometry on the basis of (A) GR1, CD11b expression and high side scatter in order to determine the effect of IL-17 neutralization on granulocyte levels in the lungs. Bacterial burdens were determined in the (B) lungs and (C) spleen of anti-IL-17- or isotype control-treated mice. Results are mean±SEM and are combined from two independent experiments, totalling ten mice *per* group except for the WT isotype-treated group in (C) where *n*=6 mice *per* group. ^*^p<0.05; ^**^p<0.01; ^***^p<0.001 (unpaired Student's *t*-test).

It has been shown 45 (and reviewed in 11) that IL-17 signalling is critical for granulocyte mobilization to the lung during *Klebsiella pneumoniae* infection. Similarly, following IL-17 neutralization, we observed a significant decrease in total numbers of lung GR-1^+^CD11b^+^ granulocytes in IL-17 Ab treated as compared with isotype control IgG-treated WT and IL-10^−/−^ mice post-*Mtb* infection (Fig. 6A). Neutralization of IL-17 had no significant effects on IFN-γ or CXCL10 production in the lung or serum, or on T-cell influx in *Mtb*-infected IL-10^−/−^ mice. Neutralization of IL-17 during primary *Mtb* infection in either WT or IL-10^−/−^ mice had no significant effect on bacterial load in the lungs at this early time point (Fig. 6B). IL-17 neutralization during primary *Mtb* infection of WT mice, and to a lesser extent IL-10^−/−^ mice, resulted in a decreased bacterial load in the spleen (Fig. 6C). The lesser effect observed in IL-10^−/−^ mice is possibly due to the higher levels of IL-17, although the bacterial load was lower to start with in the IL-10^−/−^ mice. These observations suggest that IL-17 may have adverse effects and potentially impair the host's ability to control *Mtb* infection at secondary disease sites.

## Discussion

We provide evidence that IL-10^−/−^ mice show enhanced control of *Mtb* infection with significantly reduced bacterial load in lungs and spleen, which was maintained over the course of infection studied. Increased protection in IL-10^−/−^ mice was associated with an accelerated and enhanced Th1 response in the lung. Earlier and increased levels of the cytokines/chemokines, IFN-γ, TNF, CXCL10, IL-6 and IL-17 were detected in sera and lungs of infected IL-10^−/−^ mice as compared with WT control mice, coincident with the increased and earlier influx of CD4^+^ T cells into the lungs. The earlier and enhanced production of CXCL10 and the accelerated influx of Th1 cells into the lung of infected IL-10^−/−^ mice was independent of IL-17. Instead, neutralization of IL-17 during primary *Mtb* infection resulted in a significant decrease in bacterial load in the spleen, suggesting a potential role for IL-17 in dissemination of mycobacteria.

Previous reports have suggested that IL-10^−/−^ and WT mice display identical capacity to control *Mtb* infection 23, 33, whereas others have reported enhanced early although transient protection to this pathogen in IL-10^−/−^ mice with enhanced IFN-γ production in the dLN 28. Our novel findings are distinct since we show that IL-10^−/−^ mice maintain a reduced bacterial load in the lung and spleen during *Mtb* infection, which was independent of the genetic background studied. These conflicting reports in the literature may be due to differences in the ages of mice, the initial dose of infecting bacteria and/or the microbial flora/status of mice in different laboratories. The enhanced protection that we observe in IL-10^−/−^ mice infected with *Mtb* not only correlates with early and increased responses of CD4^+^ T cells in the dLN but also with accelerated and elevated production of the chemokine CXCL10 and increased numbers of Th1 cells in the lung.

There are various potential mechanisms whereby IL-10 could suppress the immune response to *Mtb* infection, leading to reduced protection and increased bacterial burden. There is compelling evidence that the initiation of the response to *Mtb* occurs in the LN 42–44. Hence, IL-10 could function to suppress the production of cytokines/chemokines required for the migration of infected myeloid cells to the LN and thus inhibiting the initiation of a protective immune response. This is suggested by earlier findings that transferred bacillus Calmette–Guerin-infected DC from IL-10^−/−^ mice show increased trafficking to the dLN in response to mycobacterial antigens, although the effects on bacterial load were not assessed 39. In keeping with these findings, we observe an increased number of CD4^+^ T cells in the dLN of IL-10^−/−^ mice as compared with controls, although the percentages of IFN-γ-producing CD4^+^ T cells were equivalent. However, we show markedly increased percentages and numbers of IFN-γ-producing CD4^+^ T cells in the lungs of infected IL-10^−/−^ mice as compared with control mice in keeping with increased Th1 differentiation and influx into the lungs. Taken together, our data suggest that the increased numbers of IFN-γ-producing cells in the lungs result from a combination of increased activation/proliferation in the dLN, and increased migration/differentiation to the lung. In all, our findings suggest that the major effects of IL-10 to suppress the immune response to *Mtb* are in the lung and dLN, and that by neutralizing this cytokine an earlier local Th1 response in the lung can dictate the subsequent level of protection observed up to 125 days of infection. This is in keeping with the previously reported findings that enhanced protection to *Mtb* infection in relatively resistant as compared with susceptible mice is paralleled by an earlier influx of T cells producing IFN-γ 7.

The enhanced and early production of CXCL10 that we observe in *Mtb*-infected IL-10^−/−^ mice may contribute to the increased number of IFN-γ-producing CD4^+^ T cells recruited to the lungs, in keeping with the known role of CXCL10 in Th1 migration during other infections 46. Thus far, we find no apparent enhanced killing of bacilli in *ex vivo Mtb*-infected macrophages from IL-10^−/−^ mice as compared with those from WT mice (data not shown). However, it is possible that IL-10 produced by other cells during infection suppresses the production or action of microbicidal factors induced in macrophages important for protection against *Mtb* infection. Enhanced and earlier IFN-γ production, together with the increased levels of TNF, that we observed in the lungs of *Mtb*-infected IL-10^−/−^ mice will undoubtedly result in the enhanced activation of macrophages for control of mycobacterial growth, in keeping with the reported properties of these cytokines in the protective response to mycobacterial infection 4, 5, 12. Elevated levels of GM-CSF in the lung of *Mtb*-infected IL-10^−/−^ mice as compared with controls could also contribute to the enhanced protection observed, since GM-CSF has been previously shown to be important for recruitment of Th1 cells to the lung and containment of *Mtb* 47.

IL-17 has been shown to be important for the increased influx of Th1 cells into the lungs, after vaccination against *Mtb* infection 8. However, we show that neutralization of IL-17 during primary *Mtb* infection had no effect on the enhancement of IFN-γ or CXCL-10 production in the serum or lungs, nor the accelerated and enhanced numbers of Th1 cells in the lungs of IL-10^−/−^ mice. In keeping with a previous report where IL-17R-deficient mice showed no effects on *Mtb* infection 10, 11, in our study neutralization of IL-17 in either WT or IL-10^−/−^ mice showed no effect in controlling *Mtb* infection in the lung, although this was a relatively early time point. However, and quite unexpectedly, neutralization of IL-17 in WT and IL-10^−/−^ mice, resulted in a reduction in *Mtb* load in the spleen, which suggests that IL-17 may affect dissemination of mycobacteria to the spleen during an aerogenic infection. In line with this, total lung granulocyte numbers were also significantly reduced in these WT and IL-10^−/−^ anti-IL-17-treated mice as compared with isotype-treated control mice, suggesting that IL-17 through its role in granulocyte recruitment may facilitate bacterial dissemination from the lung to secondary disease sites. To support this, although dissemination of mycobacteria to dLN has been suggested to be mediated by DC 36–41, phagocytic cells of diverse phenotypes, including neutrophils have been shown to be infected with mycobacteria 38, 48, 49, suggesting a role for these cells as permissive hosts. Although it has been suggested that granulocytes may play a role in granuloma formation in relatively resistant mice 50, many reports support a negative role for neutrophils/granulocytes in TB pathogenesis in genetically susceptible mouse strains 51, 52 and in active TB patients 53, 54. During *Mtb* infection, neutrophils have recently been shown to be the dominant producers of IL-10 in the lung and depletion of these cells 55 reduced lung bacterial load while enhancing IL-6 and IL-17 but not IFN-γ responses. Collectively, these reports suggest a detrimental role for neutrophils in pathogenesis of TB.

In conclusion, we show here that IL-10 controls the primary immune response to *Mtb* infection, and that in the absence of this cytokine, mice maintain a reduced bacterial load and an accelerated and earlier Th1-type response in the lung with reduced dissemination to the spleen. Thus, it is tempting to speculate that neutralization of IL-10 during vaccination may lead to an accelerated, enhanced and nonpathological protective Th1 response against *Mtb* infection.

## Materials and methods

### Mice

Female BALB/c, BALB/c IL-10^−/−^, C57BL/6 and C57BL/6 IL-10^−/−^ were bred and housed under specific pathogen-free conditions at the MRC NIMR. CBA/J mice were purchased from Charles River, France. Experiments were in accordance with the Home Office (UK). Mice were 8–14 wk of age.

### *Mtb* infection

*Mtb* experiments were performed under Containment level-3 conditions. *Mtb* H37Rv was grown in Middlebrook 7H9 broth supplemented with 10% OADC (Difco), 0.05% Tween-80, 0.5% glycerol to mid-log phase before freezing at −80°C. For aerogenic infections, a three-jet Collision nebulizer unit (BGI, USA) was used. Briefly, 1×10^7^ CFU *Mtb* in PBS were aerosolized over a period of 15 min with approximately 30 CFU delivered to the lungs as confirmed by enumeration of bacteria on day 1 postinfection.

### *In vivo* Ab treatment

Mice were injected i.p. with 100 μg of mAb against IL-17 (R & D systems; 50104) or an isotype control IgG (R & D systems; 54447) in sterile PBS (Gibco) at the start of the *Mtb* infection (day 0), every 3–4 days until day 28, and killed at day 32 postinfection. In total, 1 mg anti-IL-10R mAb (a kind gift from DNAX, now Merck, Palo Alto, CA, USA; 1B1.3A) or isotype control IgG (Merck; GL113) was injected i.p. in PBS the day before infection and then 0.35 mg was given once weekly thereafter until the end of the experiment.

### Removal of organs for cell culture and determination of bacterial load

Spleens and lungs were aseptically removed from mice (killed by CO2 exposure) and homogenized by passing through a 70 μm sieve in cRPMI for immune function. Cell pellets were treated with 0.83% ammonium chloride, placed in culture medium, counted and restimulated *ex vivo* with either Tuberculin PPD for 48 h (10 μg/mL; Statens Serum Institut) or with PMA (50 ng/mL; Sigma-Aldrich) and ionomycin (500 ng/mL; Calbiochem) for 4 h. For intracellular IFN-γ or IL-17A analysis by flow cytometry, brefeldin A (10 μg/mL; Sigma-Aldrich) was added during the last 4 h of cell cultures. Cell suspensions were serially diluted onto 7H11 agar plates supplemented with OADC (10%) and after 18 days at 37°C, visible CFU were counted and the bacterial load *per* organ was calculated.

### Flow cytometry

For the analysis of cell surface markers, cells were pretreated for 10 min with anti-FcγRI/FcγRII (anti-CD16/CD32) to minimize a specific Ab binding. Staining was performed in Dulbecco's PBS (Gibco) supplemented with 1% FBS, penicillin (100 U/mL), streptomycin (100 mg/mL) and 0.1% sodium azide (Sigma-Aldrich). The markers CD3-APC (BD; 145-2C11), CD4-PerCP (BD; RM4-5), CD8-FITC (eBioscience; 53-6.7) and γδ TCR-PE (eBioscience; eBioGL3) were used to identify T-cell populations. Lung granulocytes were identified by the expression of GR-1-FITC (BD; RB6-8C5) and CD11b-APC (BD; M1/70) and the absence of CD11c-PE (BD; HL3) and CD4-PerCP (BD; RM4-5) and by high side scatter. Cells were stained for 30 min, washed and fixed in 2% formaldehyde (Sigma-Aldrich) before acquisition. For intracellular cytokine analysis, cells were fixed and treated with permeabilization buffer (eBioscience) according to the manufacturer's instructions and stained with IFN-γ-APC (BD; XMG1.2) and IL-17A-PE (BD; TC11-18H10) and relevantly conjugated isotype control Ab. Acquisition was performed with CellQuest software on a FACS Caliber flow cytometer (BD Biosciences) with a minimum of at least 100 000 total events being collected. The data were analysed using FlowJo version 8 (Treestar) software.

### Immunoassay/multiplex assay

Protein analysis of mouse sera and cell culture supernatants obtained from *ex vivo* restimulations was screened commercially by a “22-Multiplex cytokine profiler” assay for G-CSF, GM-CSF, IL-1α, IL-1β, IL-2, IL-4, IL-5, IL-6, IL-7, IL-9, IL-10, IL-12(p70), IL-13, IL-15, IL-17A, IFN-γ, TNF, CCL2 (MCP-1), CCL3 (MIP-1α), CCL5 (RANTES), CXCL1 (KC/Gro) and CXCL10 (IP-10) (Millipore, UK). Commercially available ELISA kits (eBioscience) were also used to determine the concentrations of IL-17A, GM-CSF and TNF in sample supernatants. ELISA assays were performed according to the manufacturer's protocol.

### Real-time PCR of *Il10* expression in lung tissue

Whole lung tissue was aseptically removed and homogenized immediately in TRI-reagent (Ambion) RNA buffer by pulsing with a Polytron PT1600E homogenizing unit (Kinematic) before being frozen at −80°C. For RNA extraction, the samples were thawed and an RNeasy kit (Qiagen) was used according to the manufacturer's protocol. RNA was reverse transcribed with a high-capacity reverse transcription kit (Applied Biosystems) to cDNA and run on an ABI PRISM 7900 sequence-detection system (Applied Biosystems). Murine *Il10* primers were used as described previously 56 and mRNA expression was quantified with SYBR Green (Applied Biosystems). Expression data shown for *Il10* were normalized to *Hprt1*.

### Statistical analysis

All data were analysed as indicated in the figure legends using Student's *t-*test (95% confidence interval) with differences being considered significant when *p*<0.05 (^*^p<0.05; ^**^p<0.01; ^***^p<0.001).

## Abbreviations

dLN: draining LN
Mtb: Mycobacterium tuberculosis
PPD: purified protein derivative
TB: tuberculosis

## Supporting information

Detailed facts of importance to specialist readers are published as ”Supporting Information”. Such documents are peer-reviewed, but not copy-edited or typeset. They are made available as submitted by the authors.
