# Supplementary material for: Enhanced protection to Mycobacterium tuberculosis infection in IL-10-deficient mice is accompanied by early and enhanced Th1 responses in the lung
Source: Eur J Immunol. 2010 Jun 1;40(8):2200–10. doi: 10.1002/eji.201040433 (PMC3378704; doi:10.1002/eji.201040433)
Supplement: Supplementary file 1 [file eji0040-2200-SD1.pdf]

European Journal of Immunology

Supporting Information

for

DOI 10.1002/eji.201040433

Enhanced protection to *Mycobacterium tuberculosis* infection in IL-10-deficient mice is accompanied by early and enhanced Th1 responses in the lung

Paul S. Redford, Andre Boonstra, Simon Read, Jonathan Pitt, Christine Graham, Evangelos Stavropoulos, Gregory J. Bancroft and Anne O'Garra

© 2010 WILEY-VCH Verlag GmbH & Co. KGaA, Weinheim

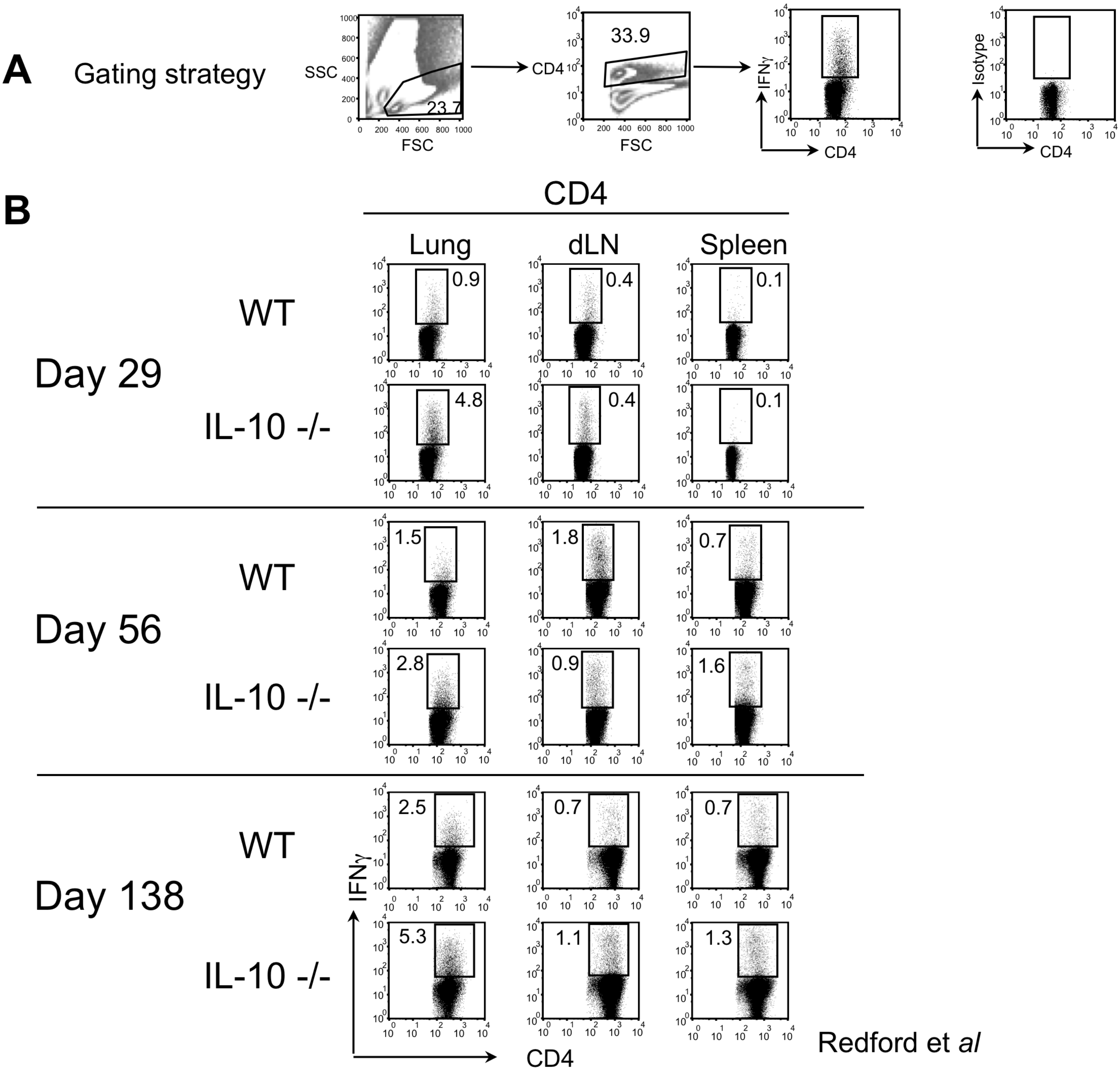

Redford et *al*

Supporting information Figure 1: IL-10-/- mice have early enhanced percentages of IFN-γ$^+$ CD4+ cells in the lungs.

Whole organ homogenates from *M. tuberculosis* infected wild type BALB/c and BALB/c IL-10-/- mice were stimulated *ex vivo* with PPD for 48 hours before cells were stained for flow cytometric analysis. (A) The gating strategy to determine the percentage production of IFN-γ from CD4$^+$ cells along with an appropriately labelled isotype control antibody for IFN-γ. (B) Percent CD4$^+$IFN-γ$^+$ cells present in the lungs, dLN and spleen at the indicated time-points post *M. tuberculosis* infection. FACS plots shown are concatenations of 3-4 individual mice per group per time-point and are representative of at least 2 independent experiments.

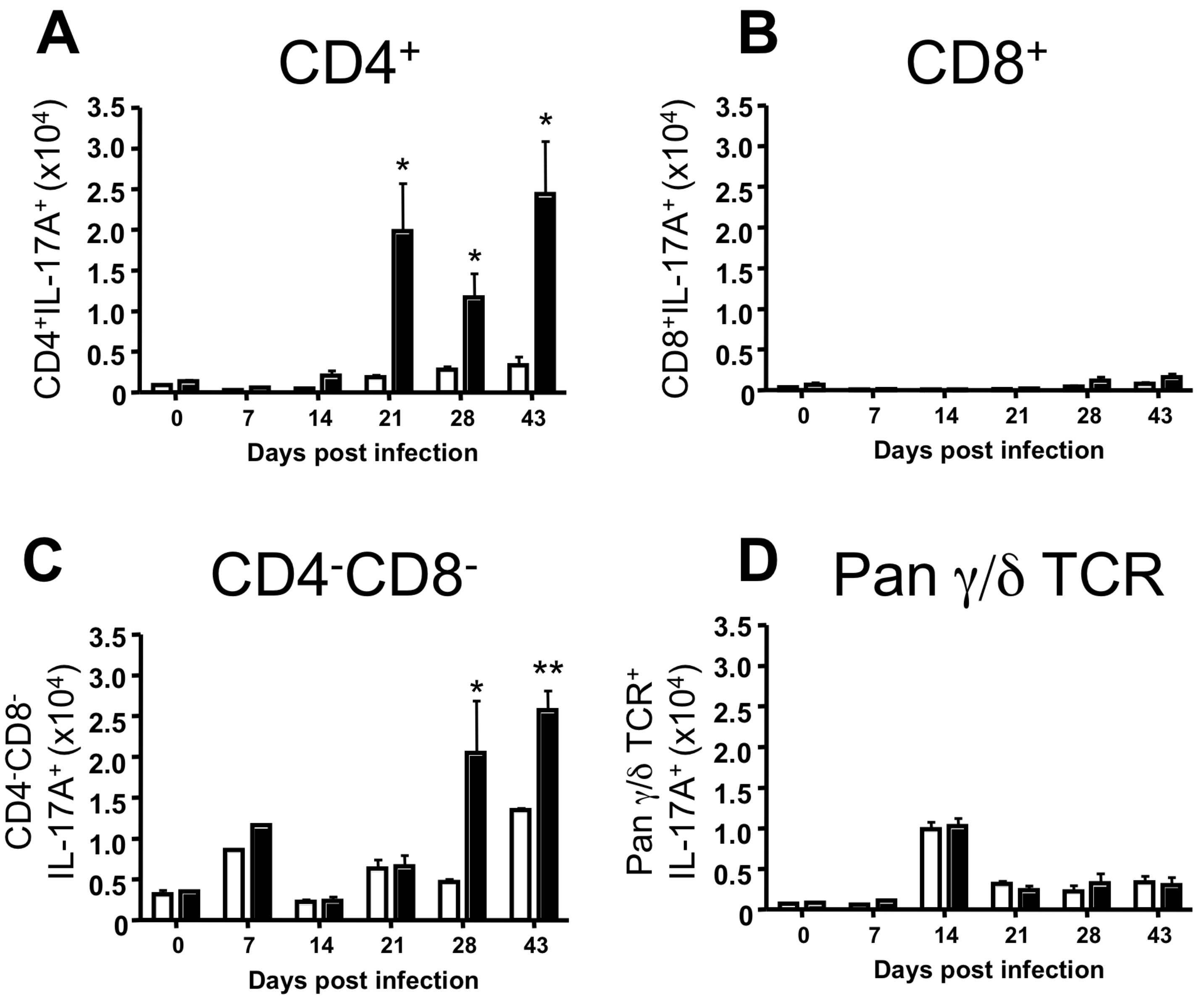

Redford *et al*

Supporting information Figure 2: The proportion of IL-17A+ cells is enhanced early in MTb infected IL-10-/- mice.

Wild type BALB/c (open bars) and BALB/c IL-10-/- (closed bars) mice were infected with *M. tuberculosis* as described in Fig. 1, and whole lung homogenates were restimulated with PMA and Ionomycin as described in Materials and Methods. IL-17A production from (A) $CD4^+$; (B) $CD8^+$; (C) $CD4^-CD8^-$ (D) g/d T cells was analysed by flow cytometry as described according to the gating strategy in Fig. S1 A. Total numbers of IL-17A-producing cells of each subset are shown from at least 4 individual mice per group per time-point. The effect attributed to the absence of IL-10 was tested statistically using an unpaired Students *t* test (*, $P < 0.05$; **, $P < 0.01$; ***, $P < 0.001$).

Supporting information Table 1: Preliminary results from an initial screen of Lung supernatants by Multiplex analysis from uninfected and *Mtb* infected (day 28-29) wild type and IL-10-/- mice (BALB/c background).

Analyte:	Independent Expts.	WT non-infected	IL-10-/- non-infected	WT *Mtb* day 28	IL-10-/- *Mtb* day 28
IL-1α	Expt. 1	3.523 ± 1.361	10.53 ± 2.770	29.58 ± 6.124	127.6 ± 30.28
IL-1α	Expt. 2	12.47 ± 5.694	11.63 ± 1.807	90.03 ± 4.319	127.2 ± 5.053
IL-1β	Expt. 1	1.028 ± 0.6507	3.030 ± 0.3233	13.70 ± 1.485	44.53 ± 9.032
IL-1β	Expt. 2	4.822 ± 2.009	4.647 ± 0.8207	13.12 ± 2.515	16.77 ± 1.839
IL-2	Expt. 1	<<	<<	6.550 ± 1.728	37.90 ± 16.59
IL-2	Expt. 2	<<	<<	8.090 ± 0.3749	50.25 ± 15.96
IL-4	Expt. 1	<<	<<	1.650 ± 0.02887	3.525 ± 0.6860
IL-5	Expt. 1	8.985 ± 2.620	6.400 ± 0.3559	36.50 ± 12.50	21.18 ± 3.886
IL-7	Expt. 1	1.018 ± 1.018	3.125 ± 3.125	8.643 ± 2.613	11.90 ± 2.041
IL-9	Expt. 1	<<	<<	17.28 ± 6.212	35.70 ± 5.431
IL-9	Expt. 2	1.475 ± 1.475	1.573 ± 1.573	<<	1.907 ± 1.436
IL-12(p70)	Expt. 1	<<	<<	4.238 ± 1.780	9.350 ± 1.883
IL-13	Expt. 1	4.135 ± 0.3893	5.750 ± 0.9526	13.60 ± 2.305	23.08 ± 3.175
IL-15	Expt. 1	<<	<<	<<	<<
CCL2 (MCP1)	Expt. 1	842.8 ± 185.7	505.5 ± 61.26	454.8 ± 59.41	239.3 ± 26.94
CCL2 (MCP1)	Expt. 2	317.8 ± 39.68	211.2 ± 35.20	530.7 ± 60.66	149.0 ± 13.48
CCL3 (MIP1α)	Expt. 1	174.2 ± 9.283	263.3 ± 14.43	719.3 ± 137.5	1139 ± 246.9
CCL3 (MIP1α)	Expt. 2	116.7 ± 13.47	210.3 ± 24.94	841.8 ± 111.8	841.7 ± 184.8
CCL5 (RANTES)	Expt. 1	4.525 ± 0.3010	3.175 ± 0.2394	41.30 ± 7.208	79.15 ± 17.83
CXCL1 (KC/GRO)	Expt. 1	1838 ± 283.5	2176 ± 52.38	2534 ± 268.9	4079 ± 638.2

a) All Means shown are ± S.E.M. (as outlined in Figure 3).
b) << = below detection (<1pg/ml).
c) Expt.1 = preliminary screen; Expt.2 = repeat of selected analytes to confirm initial findings.

Supporting information Table 2: Preliminary results from an initial screen of serum samples by Multiplex analysis from uninfected and *Mtb* infected (day 28) wild type and IL-10-/- mice (BALB/c background).

	**WT**	**IL-10-/-**	**WT**	**IL-10-/-**
**Analyte:**	**non-infected**	**non-infected**	***Mtb* day 28**	***Mtb* day 28**
IL-1α	28.9 ± 2.9	25.9 ± 6.65	41.8 ± 10.3	36 ± 9.22
IL-1β	<<	9.4 ± 0.37	<<	9.4 ± 5.79
IL-2	<<	<<	<<	<<
IL-4	<<	<<	<<	<<
IL-5	4.9 ± 1.3	2.8 ± 0.4	6.3 ± 1.5	8.5 ± 5.3
IL-7	9.4 ± 8.2	5.1 ± 3.3	12.1 ± 10.6	2.4 ± 1.7
IL-9	<<	11.7 ± 3	50.8 ± 26	<<
IL-10	<<	<<	<<	<<
IL-12(p70)	33.5 ± 1.5	25 ± 6.2	6.3 ± 1.8	18.7 ± 5.02
IL-13	12.0 ± 1.8	11.9 ± 1.9	13 ± 1.7	11.3 ± 1.7
IL-15	98.7 ± 38.6	186.3 ± 51.6	179.8 ± 39	51.9 ± 8.8
GM-CSF	44.7 ± 14.5	23.7 ± 16.76	10.9 ± 6.4	24.4 ± 3.7
CCL2 (MCP1)	18.5 ± 6.2	27.9 ± 7	13.4 ± 2.7	74.1 ± 19.1
CCL3 (MIP1α)	22.5 ± 3.8	20.2 ± 2.6	15.7 ± 3.8	21.3 ± 4.6
CCL5 (RANTES)	7.3 ± 1.6	9.8 ± 2.1	6.4 ± 0.9	13.7 ± 7.5
CXCL1 (KC/GRO)	31.5 ± 16.4	47.8 ± 27.6	17.4 ± 6.6	56.4 ± 22.4

a) All Means shown are ± S.E.M. (as outlined in Figure 4)
b) << = below detection (<1pg/ml)
